# Using a model filamentous fungus to unravel mechanisms of lignocellulose deconstruction

**DOI:** 10.1186/1754-6834-6-6

**Published:** 2013-01-22

**Authors:** Elizabeth A Znameroski, N Louise Glass

**Affiliations:** 1 Department of Molecular and Cell Biology, University of California, 94720, Berkeley, CA, USA; 2 Current address: Novozymes, 1445 Drew Avenue, 95618, Davis, CA, USA; 3 Department of Plant and Microbial Biology, University of California, 94720, Berkeley, CA, USA

**Keywords:** Cellulase, Lignocellulosic biofuels, Filamentous fungus, Transcriptome, Secretome, Neurospora, Trichoderma

## Abstract

Filamentous fungi are the main source of enzymes used to degrade lignocellulose to fermentable sugars for the production of biofuels. While the most commonly used organism for the production of cellulases in an industrial setting is *Trichoderma reesei* (*Hypocrea jecorina*), recent work in the model filamentous fungus *Neurospora crassa* has shown that the variety of molecular, genetic and biochemical techniques developed for this organism can expedite analyses of the complexities involved in the utilization of lignocellulose as a source of carbon. These include elucidating regulatory networks associated with plant cell wall deconstruction, the identification of signaling molecules necessary for induction of the expression of genes encoding lignocellulolytic enzymes and the characterization of new cellulolytic enzymatic activities. In particular, the availability of a full genome deletion strain set for *N. crassa* has expedited high throughput screening for mutants that display a cellulolytic phenotype. This review summarizes the key findings of several recent studies using *N. crassa* to further understanding the mechanisms of plant cell wall deconstruction by filamentous fungi.

## Background

Every year photosynthesis converts approximately 100 billion metric tons of CO_2_ and H_2_O into cellulose [1]. Cellulose, the β-(1,4)-linked glucose polymer, is the most abundant biomolecule on the planet and provides structural support to plant cell walls (lignocellulose). Plant biomass is widely viewed as a potential feedstock for the production of liquid biofuels [2]. However, a major barrier to mass production of biofuels is the inability to efficiently convert insoluble polysaccharides to fermentable sugars [3]. Both ascomycete and basidiomycete filamentous fungi have the capacity to secrete large amounts of lignocellulosic enzymes that release fermentable sugars from plant cell walls.

The most commonly used organism for the production of cellulases in an industrial setting is the filamentous ascomycete species *Trichoderma reesei* (*Hypocrea jecorina*) [4]. While this organism was selected for its ability to secrete plant cell wall degrading enzymes, it has since undergone several rounds of random mutagenesis, resulting in industrial strains that secrete cellulase quantities exceeding 100 gL of culture [5]. With the availability of low cost and high-throughput sequencing methods, studies have begun to shed light on mutations that may be relevant for the cellulase hyperproduction phenotype [6]. Although over 200 mutations were identified, the genetic, molecular and biochemical techniques to unravel the role of these mutations in the hypercellulolytic industrial *T. reesei* strains are somewhat problematic due to lack of extensive genetic and molecular tools.

**Figure 1:**
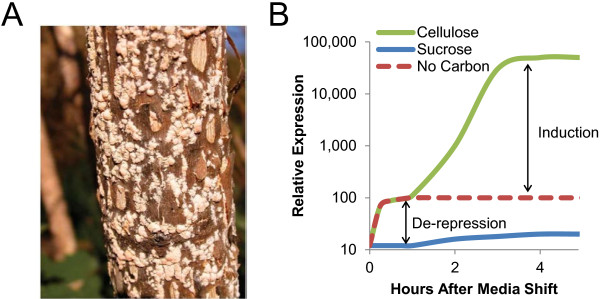
**Growth of *N. crassa *on lignocellulose.** (**A**) Photo of *N. crassa* growing on a burnt tree in Seville, Spain in 2004 [13]. (**B**) Characteristic expression pattern of lignocellulose degrading enzymes when grown in a media containing cellulose (green), sucrose (blue), or no carbon (red).

The related ascomycete species, *Neurospora crassa*, is proficient at degrading lignocellulose for use as a source of carbon. In its natural environment, *N. crassa* lives mainly in tropical and sub-tropical regions where it can be found growing on dead plant matter after fires (Figure 1A). Cellulases in *N. crassa* were identified as early as 1949 [7] and Bruce Eberhart first began characterizing them in the late 1970s [8]. Almost nothing further was reported until a recent systems analysis of *N. crassa* grown on a potential biofuel substrate, the perennial grass *Miscanthus*[9]. *N. crassa* has the unique advantage of being a National Institutes of Health (NIH) model organism, most commonly known for its role proving the “one gene, one enzyme” hypothesis [10]. The genome of *N. crassa* was the first characterized for any filamentous fungus, and, similar to other genomes from this group of organisms, ~40% of its genes are of unknown function [11]. Importantly, a variety of molecular, genetic and biochemical techniques have been developed for *N. crassa*, including a publically available full genome deletion strain set [12].

Similar to other filamentous fungi, *N. crassa* induces lignocellulolytic enzymes in response to exposure to plant cell wall material [9]. Using transcriptomics, proteomics and functional genomics approaches, recent studies on *N. crassa* have begun to shed light on the mechanisms required for *N. crassa* to go from growth on a simple sugar to a much more complex and recalcitrant molecule such as cellulose. Given the high conservation of lignocellulose degrading machinery in filamentous fungi, the knowledge gained by studying *N. crassa* will shed light on mechanisms important for the industrial production of lignocellulose degrading enzymes and biofuels. A specific example of this translation to industrial relevant studies occurred when cellodextrin transporters and an intracellular β-glucosidase (identified via a transcriptomics approach in *N. crassa*[9]) were transferred to *Saccharomyces cerevisiae,* increasing its capacity to utilize cellodextrins with a concomitant increase in ethanol yield [14].

### Expression of cellulases and hemicellulases in *N. crassa* in response to insoluble lignocellulose

The *N. crassa* genome (39.9 Mb) contains approximately 10,000 genes [11,15] and is predicted to contain twice as many cellulases as *T. reesei*, as well as many hemicellulases and other enzymes involved in plant biomass degradation [16]. To examine the response of *N. crassa* to lignocellulose, a systems analysis used microarray expression data to identify ~800 genes that varied in expression level when *N. crassa* was grown on *Miscanthus* over a 10 day time period as compared to expression levels when grown for 16 hours on sucrose [9]. More recently, next generation RNA sequencing was used to assess the early induction mechanisms of *N. crassa* in response to Avicel (crystalline cellulose), as compared to sucrose or no carbon source [17,18]. These studies indicate that the initial response of *N. crassa* to a cellulosic substrate is a relief from carbon catabolite repression (de-repression), followed by an induction phase in response to the cellulosic signal (Figure 1B). Carbon catabolite repression has been described in detail elsewhere [19,20], however, in brief, the phenomenon allows an organism to utilize a preferred (easily metabolized) carbon source by actively inhibiting the expression of genes required to metabolize more complex sources of carbon. The de-repression stage is characterized by a small increase in expression level of genes encoding cellulases, with similar levels of expression seen in *N. crassa* cultures transferred to media containing no carbon source or media containing Avicel as the sole carbon source. In the no carbon source cultures, the expression of cellulose responsive genes do not show any further increase in expression, while in Avicel cultures, this set of genes is induced several orders of magnitude further. Thus, similar to other filamentous fungi, full induction of lignocellulose degrading enzymes in *N. crassa* requires the presence of specific inducing molecules.

The disaccharide cellobiose (two glucose molecules linked by a β(1→4) bond) is the major soluble end product of cellulase activity [21]. Growth on cellobiose induces low levels of cellulase gene expression and activity in *T. reesei*[22] and *Aspergillus* species [23], but does not induce cellulases in *N. crassa*[17] or *Phanerochaete chrysosporium*[24]. The oligosaccharide sophorose, which can be generated by the transglycosylation of cellobiose by β-glucosidases, acts as a potent inducer of cellulases in *T. reesei*[25], but not in *N. crassa* or other filamentous fungi [17,26]. The main enzymatic activity of a β-glucosidase is to hydrolyze cellodextrins to glucose; glucose represses cellulase gene expression in filamentous fungi due to carbon catabolite repression [27]. In *N. crassa*, a strain carrying deletions for the three major β-glucosidase genes (thus preventing almost all conversion of cellobiose into glucose) results in the induction of cellulases in response to cellobiose [17]. This induction of genes/enzymes recapitulates, on a transcriptional and protein level, the wild type response to crystalline cellulose. Similar work in *T. reesei* suggests that cellobiose, and not sophorose, may be the natural inducing molecule in this species [28]. While data provided by Zhou and colleagues [28] showed that absence of the major β-glucosidases compromised efficient induction of cellulases when cultured on cellulose, this mutant strain showed increased transcription of cellulases in response to cellobiose. Thus, in both *N. crassa* and *T. reesei,* these β-glucosidase enzymes are not required for efficient cellulase induction. While the inducer cannot be conclusively defined as cellobiose, both of these studies suggest that the hypothesis that a transglycosylated form of cellobiose (sophorose) produced by β-glucosidase enzymes [25,29,30] is the signal molecule required for efficient cellulose induction in nature is probably not correct.

**Figure 2:**
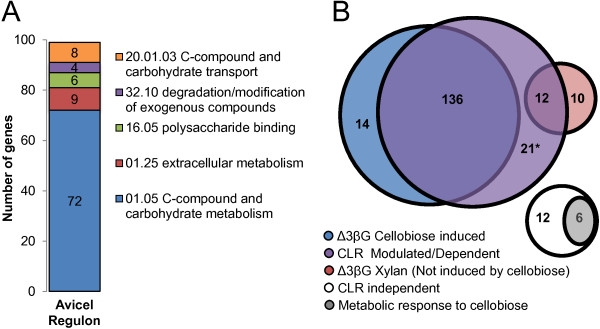
**Analysis of genes induced when *N. crassa *is grown on cellulose.** (**A**) Functional category analysis of genes in the Avicel regulon [18]. (**B**) Venn diagram for the cellobiose/Avicel and CLR regulons. Genes were divided into the following categories: blue: cellobiose induction, purple: CLR mediated or dependent, white: CLR independent, grey: metabolic response to cellobiose. *21 gene set includes the three β-glucosidase genes which were not included in the cellobiose analysis [17]. Complete data set is available as Additional file 1.

In *N. crassa*, only 333 genes are significantly differentially expressed when cultures are transferred from sucrose to media lacking any carbon source compared to cultures shifted to media containing Avicel [17,18]. Of these 333 genes, 212 show increased expression in response to Avicel and are considered the “Avicel regulon” (Additional file 1). More than 50% of the proteins encoded by these 212 genes are predicted to enter the secretory pathway [18]. In addition, 99 genes are predicted to encode proteins involved in the degradation and use of carbon compounds (Figure 2A), approximately 60 of which are identified by the CAZy database as glycoside hydrolases [31]. Included in this set are 17 of 21 predicted cellulase and 11 of 19 predicted hemicellulase genes in the *N. crassa* genome [9]. Importantly, 88 genes within the Avicel regulon encode proteins that are annotated as “hypothetical”, and lack data indicating a biochemical function [18]. Using the *N. crassa* full genome deletion set, cellulolytic and/or hemicellulolytic phenotypes were identified for strains carrying deletions of some of these genes [9,32], however the biochemical or cellular function of most of these “hypothetical” proteins/genes have yet to be analyzed. Given that these genes are specifically induced under cellulolytic conditions, they represent an excellent set to further characterize for their involvement in plant biomass deconstruction.

Using a ~200 transcription factor deletion strain set available for *N. crassa*[33], two conserved transcription factors (CLR-1 and CLR-2) were identified that are required for expression of cellulases and growth on crystalline cellulose, but are not required for growth on xylan [18]. The function of *clr-2* in regulating cellulase gene expression is conserved in the distantly related filamentous ascomycete fungus, *Aspergillus nidulans*[18]. Comparison of the CLR-1/CLR-2 regulon with the cellobiose induced gene set shows that 136 genes are induced when *N. crassa* is exposed to Avicel or cellobiose (in the β-glucosidase mutant) (Figure 2B). These genes form the core group of genes within the Avicel regulon, including 16 of the 17 cellulases and 7 of the 11 hemicellulases, as well as several genes encoding cellodextrin transporters and genes that are likely involved in degrading more complex sources of lignocellulose including mannan, pectin and arabinose. Finally, this group contains 55 hypothetical proteins, whose role in cellulose deconstruction has yet to be examined.

A second group contains 33 genes that are modulated or dependent on CLR-1/CLR-2, but are not significantly induced by cellobiose in the β-glucosidase mutant strain (Figure 2B). Notably, this set includes 12 genes that are induced by Avicel, but not by cellobiose in the β-glucosidase mutant strain, indicating that while these genes are affected by CLR-1 and/or CLR-2, their expression is likely affected by other factors. For example they may be under more complex regulation such as carbon catabolite repression, other signals, or possibly CLR-1/CLR-2 may regulate genes in addition to those directly involved in cellulose degradation. The third group contains 10 genes that are CLR-1/CLR-2 independent, but are induced by Avicel. This gene set includes those induced due to hemicellulose contamination of Avicel [18]. The remaining groups of genes include those that are CLR independent, however, they do include several genes predicted to encode enzymes associated with plant cell wall degradation. Finally, 6 genes fall into the category of “metabolic response to cellobiose”. These genes were classified previously [17] as they are induced when WT is transferred either to Avicel or high concentrations of cellobiose. These genes are regulated independently of CLR-1 and/or CLR-2 and none have specific annotation, leading us to hypothesize that they may be involved in the metabolic response to cellobiose. Altogether, these data indicate that CLR-1/CLR-2 are essential for regulating the majority of genes in the Avicel regulon, but that other transcription factors may also play a role in cellulose deconstruction.

In addition to CLR-1 and CLR-2, *N. crassa* also contains a homolog of *xlnR* (*xlr-1*), another transcription factor involved in growth on lignocellulosic biomass. While work in both *N. crassa*[32] and *Fusarium*[34,35] species indicates that the genes regulated by XLR-1/XlnR are limited to those required to degrade xylan, in *T. reesei* and *Aspergillus* species, XYR1/XlnR regulates the expression of both cellulase and hemicellulose genes [36-38]. These studies suggest that some fungal species co-regulate the expression of cellulose and hemicellulase genes, while other species evolved independent regulatory mechanisms. However, in *A. nidulans*, the *clr-2* homolog, *clrB*, is absolutely required for cellulolytic activity [18]; *clr-2* orthologs are present in all filamentous ascomycete fungal genomes that are capable of degrading plant cell wall material, including *T. reesei.* We expect that further comparisons of the CLR-1/CLR-2 and XlnR regulons in diverse filamentous fungi will define conserved and divergent features associated with regulation of genes involved in plant cell-wall deconstruction. Further work on comparative mechanistic details on the regulation of cellulase and hemicellulase genes will provide details needed to understand co-regulation versus independent regulation of lignocellulolytic genes and may provide clues to the evolutionary advantage/disadvantage of different regulatory modes.

### The secretome of *N. crassa* when grown on cellulose

In addition to studies performed on the transcriptome of *N. crassa* in response to lignocellulose, proteomic approaches identified secreted proteins when *N. crassa* is grown on *Miscanthus* and Avicel [9]. Quantitative proteomic approaches showed that only 13 proteins comprise 91% of the of the total *N. crassa* secretome (weight percent of supernatant) in response to crystalline cellulose [39]. In addition, 65% of the secretome is made up of 4 proteins (CBH-1, GH6-2, GH5-1, and GH3-4; Table 1). This core set of three cellulases and a β-glucosidase is highly conserved among a diverse set of lignocellulose degrading filamentous fungi, such as *T. reesei*, *A. nidulans* and *P. chrysosporium* (Table 2). While comparable quantitative mass spectrometry data is not available, recent studies indicate that this core set of enzymes is secreted by diverse filamentous fungi in response to various sources of lignocellulosic biomass [40-42], implying that the basic mechanism for lignocellulose degradation is highly conserved. The synergistic action of these enzymes has been well established and defines the classical scheme for cellulose degradation: (1) GH5 enzymes are endo-1,4-β-glucanases that randomly cleave internal bonds in the cellulose chain; (2) GH7 and GH6 enzymes are exo-1,4-β-glucanases or cellobiohydrolases that cleave cellobiose molecules from either the reducing or non-reducing ends of a cellulose chain; and (3) GH3 enzymes are β-glucosidases that hydrolyze cellobiose into two glucose molecules. However, by reconstituting these 4 purified proteins (CBH-1, GH6-2, GH5-1 and GH3-4) in identical stoichiometry to the *N. crassa* secreted protein mixture, only 43% of the total cellulase activity was recapitulated, even though these 4 proteins comprise 65% of the culture supernatant by weight [39]. These results imply that proteins other than these four canonical fungal cellulases play an important role in cellulose degradation.

**Table 1:** **Nomenclature of cellulolytic enzymes in *N. crassa *and *T. reesei***

**Function**	***N. crassa***^**a**^	***T. reesei***^**b**^	**CAZy family**^**c**^
Cellobiohydrolase	CBH-1	CBH1/CEL7A	GH7
Cellobiohydrolase	GH6-2	CBH2/CEL6	GH6
Endoglucanase	GH5-1	EG2/CEL5A	GH5
Endoglucanase	GH7-1	EG1/CEL7B	GH7
Endoglucanase	GH6-3	ND	GH6
β-glucosidase	GH3-4	BGL1/CEL3A	GH3
Lactonase	CDH-1	ND	CBM1
Polysaccharide monooxygenase	GH61-1	ND	GH61
Polysaccharide monooxygenase	GH61-2	ND	GH61
Polysaccharide monooxygenase	GH61-5	ND	GH61

ND: No significant homolog found in organism; ^a^From *Neurospora crassa* e-Compendium: http://www.bioinformatics.leeds.ac.uk/~gen6ar/newgenelist/genes/gene_list.htm, ^b^From *T. reesei* genome database: http://genome.jgi-psf.org/Trire2/Trire2.home.html, ^c^Cantarel et al., [31].

**Table 2:** **Identification of key cellulases in *N. crassa*, *T. reesei*, *A. nidulans *and *P. chrysosporium***

**Annotation**	***N. crassa *GeneID**	***N. crassa *total secreted mass percentage**^**a**^	***T. reesei *GeneID**^**b**^	**Identified in Proteome**^**c**^	***A. nidulans *GeneID**^**d**^	**Identified in Proteome**^**e**^	***P. chrysosporium *GeneID**^**f**^	**Identified in Proteome**^**g**^
CBH-1	NCU07340	39.5%	123989	+	AN00494	+	129310	+
GH6-2	NCU09680	13.4%	72567	+	ND		133052	+
GH61-2	NCU07898	6.6%	ND		ND		10320	-
GH5-1	NCU00762	5.9%	120312	+	AN01285	+	4361	+
GH61-5	NCU08760	4.6%	ND		AN01602	-	75823	-
GH7-1	NCU05057	4.0%	122081	+	AN03418	+	129310	+
GH61-1	NCU02240	3.4%	ND		ND		41650/41563	-
GH6-3	NCU07190	3.2%	ND		ND		ND	+
GH3-4	NCU04952	3.8%	76672	+	AN02828	+	139063	+
CDH-1	NCU00206	2.4%	ND		AN07230	+	11098	+

ND: No significant ortholog found in organism.

^a^Phillips, et al., [39], ^b^From *T. reesei* genome database: http://genome.jgi-psf.org/Trire2/Trire2.home.html, ^c^Jun, et al., [40]^d^From *A. nidulans* genome database: http://www.aspgd.org, ^e^Saykhedkar, et al., [41]^f^From *P. chrysosporium* genome database: http://genome.jgi.doe.gov/Phchr1/Phchr1.home.html, ^g^Adav et al., [42].

After GH5-1, CBH-1, GH6-2 and GH3-4, the next most abundant group of proteins in the *N. crassa* cellulose secretome are three GH61 proteins (15%). Until 2011, GH61 proteins were classified as endoglucanases, due to a report of a *T. reesei* GH61 that showed weak endoglucanase activity [43]. However, recent work in both *N. crassa* and in *Thermoascus aurantiacus* showed that GH61 proteins encode a novel class of copper dependent enzymes that catalyze the oxidative cleavage of cellulose in the presence of an external electron donor, and which are now referred to polysaccharide monooxygenases (PMOs) [44,45]. As above, the work in *N. crassa* was aided by the availability of gene deletion strains and genetic/expression tools that aided purification and characterization of the PMOs. While the specific catalytic mechanism is the subject of current research (see [46]), initial data suggests that in nature GH61 enzymes may receive electrons from the action of cellobiose dehydrogenase (CDH) [47], which makes up 2.4% of the *N. crassa* culture cellulose secretome by weight. Interestingly, while both GH61 proteins and a CDH were identified in the cellulose-induced secretome of *N. crassa*, orthologous proteins show variable conservation in the secretomes of related fungi (Tables 1, 2). For example, while orthologs of *N. crassa* genes encoding GH61 enzymes and a CDH can be identified in the genomes of *A. nidulans* and *P. chrysosporium*, orthologs are not clearly identifiable in the genome of *T. reesei*. While traditional industrial cellulase cocktails have been primarily composed of cellobiohydrolases, endoglucanases and β-glucosidases, the inclusion of GH61s/CDH enzymes should increase the efficiency of cellulose deconstruction. The identification and characterization of the PMOs further strengthens the idea that by investigating mechanisms beyond the traditional understanding of cellulose degradation, it may be possible to capitalize on the diversity of fungal enzymes for more efficient conversion of plant biomass.

### Concluding remarks

Historically, work on lignocellulose degrading filamentous fungi has primarily focused on *T. reesei* because of its importance in industry and many of these studies have focused on improving the enzyme yield when growing under industrially relevant conditions. However, our understanding of the interwoven regulatory networks associated with plant cell wall deconstruction (cellulose, hemicellulose, pectin and other associated carbohydrates moieties), transport and other enzymatic functions is still fragmented, as is how lignocellulose degrading filamentous fungi adapt and respond to a rapidly changing environment during plant biomass deconstruction. The molecular, biochemical and genetic tools available for *N. crassa* make it an attractive model for dissecting the deconstruction of lignocellulose, and include high throughput screening using the complete genome deletion set, the ease of the construction of strains containing multiple mutations by genetic methods [48], as well as RNA sequencing to identify sets of genes that respond to plant cell wall components, and proteomics and biochemical assays to elucidate the function, mechanism and structure of proteins associated with plant cell wall deconstruction. The results of recent studies on plant cell wall deconstruction using *N. crassa* show how the use of a developed model organism can be highly beneficial for scientific understanding of an industrially relevant process. However, given the diversity of fungi capable of degrading lignocellulose, it is probable that new mechanisms and enzymes await discovery, as recently shown by analyses of the genome of the brown rot fungus, *Postia placenta*[49]. The examination and elucidation of a diverse set of mechanisms associated with plant cell wall deconstruction could enhance tailored development of new cost-effective lignocellulose based biofuels.

## Abbreviations

CAZy: Carbohydrate-active enzymes database;CLR: Cellulose degradation regulator;XLR/XlnR: Xylan degradation regulator;CBH: Cellobiohydrolase;GH: Glycoside hydrolase;PMO: Polysaccharide monooxygenase;CDH: Cellobiose dehydrogenase;BGL: β-glucosidase;CBM: Carbohydrate binding module;ND: None determined

## Competing interests

This work was funded by the an Energy Biosciences Institute grant to N.L.G.. Published applications include: methods and compositions for improving sugar transport, mixed sugar fermentation, and production of biofuels WO2011011796.

## Supplementary Material

Additional file 1 **Analysis of genes induced when *N. crassa *is grown on cellulose.** Click here for file
